# Inflammatory-Metal Profile as a Hallmark for COVID-19 Severity During Pregnancy

**DOI:** 10.3389/fcell.2022.935363

**Published:** 2022-08-09

**Authors:** Johana Vásquez-Procopio, Aurora Espejel-Nuñez, Johnatan Torres-Torres, Raigam Jafet Martinez-Portilla, Salvador Espino Y. Sosa, Paloma Mateu-Rogell, Veronica Ortega-Castillo, Maricruz Tolentino-Dolores, Otilia Perichart-Perera, José Osman Franco-Gallardo, José Alberto Carranco-Martínez, Scarleth Prieto-Rodríguez, Mario Guzmán-Huerta, Fanis Missirlis, Guadalupe Estrada-Gutierrez

**Affiliations:** ^1^ Department of Immunobiochemistry, Instituto Nacional de Perinatología, Mexico City, Mexico; ^2^ Department of Physiology, Biophysics and Neuroscience, Center for Research and Advanced Studies (Cinvestav), Mexico City, Mexico; ^3^ Clinical Research Division, Instituto Nacional de Perinatología, Mexico City, Mexico; ^4^ Department of Obstetrics, Instituto Nacional de Perinatología, Mexico City, Mexico; ^5^ Department of Nutrition and Bioprogramming, Instituto Nacional de Perinatología, Mexico City, Mexico; ^6^ Data Science Department, Openlab, Mexico City, Mexico; ^7^ Department of Translational Medicine, Instituto Nacional de Perinatología, Mexico City, Mexico; ^8^ Research Division, Instituto Nacional de Perinatología, Mexico City, Mexico

**Keywords:** SARS – CoV – 2, placenta, zinc, copper, IL-6

## Abstract

Pregnancy makes women more susceptible to infectious agents; however, available data on the effect of SARS-CoV-2 on pregnant women are limited. To date, inflammatory responses and changes in serum metal concentration have been reported in COVID-19 patients, but few associations between metal ions and cytokines have been described. The aim of this study was to evaluate correlations between inflammatory markers and serum metal ions in third-trimester pregnant women with varying COVID-19 disease severity. Patients with severe symptoms had increased concentrations of serum magnesium, copper, and calcium ions and decreased concentrations of iron, zinc, and sodium ions. Potassium ions were unaffected. Pro-inflammatory cytokines IL-6, TNF-α, IL-8, IL-1α, anti-inflammatory cytokine IL-4, and the IP-10 chemokine were induced in the severe presentation of COVID-19 during pregnancy. Robust negative correlations between iron/magnesium and zinc/IL-6, and a positive correlation between copper/IP-10 were observed in pregnant women with the severe form of the disease. Thus, coordinated alterations of serum metal ions and inflammatory markers – suggestive of underlying pathophysiological interactions—occur during SARS-CoV-2 infection in pregnancy.

## Introduction

Pregnant women and their neonates are considered vulnerable populations in the coronavirus disease 2019 (COVID-19) pandemic, caused by the severe acute respiratory syndrome coronavirus 2 (SARS-CoV-2) (Khoury et al., 2020; Knight et al., 2020; Teles Abrao Trad et al., 2020; Valdespino-Vázquez et al., 2021). Although most pregnant women infected with SARS-CoV-2 do not show clinical symptoms (Adhikari et al., 2020; Maru et al., 2020; Cardona-Pérez et al., 2021; Luo et al., 2021; Salem et al., 2021) some develop severe/critical disease (Garcia-Flores et al., 2022). In Mexico, 30,370 pregnant women had been infected with SARS-CoV-2 from June 2020 until October 2021, of which 629 succumbed to the disease, according to the official report by the Ministry of Health (Secretaría de Salud, 2021). Indeed, during this period, the most common cause of maternal death was COVID-19 (Secretaría de Salud, 2021). Although placental COVID-19 infection has been documented in some cases (Hosier et al., 2020; Schwartz and Morotti, 2020; Vivanti et al., 2020), whether transmission occurs from the mother to the fetus and how the mother-baby dyad is affected during SARS-CoV-2 infection remain controversial issues (Mirbeyk et al., 2021; Salem et al., 2021; Sevilla-Montoya et al., 2021; Garcia-Flores et al., 2022).

Since the beginning of the pandemic, it was determined in the general population that patients with severe forms of infection exhibit elevated levels of inflammatory cytokines, such as IL-6 and TNF-α, associated with end-organ damage and lethality (Chen G. et al., 2020; Mandel et al., 2020; Pedersen and Ho, 2020). Some studies have suggested that critical changes in the immune response, including cytokine levels, occur in pregnant women with COVID-19 varying according to pregnancy trimesters and seem to be correlated with the clinical outcomes (Chen et al., 2021a; Chen et al., 2021b; Tanacan et al., 2021).

Besides inflammatory cytokines, serum metal ions have also been considered potential contributors to COVID-19 severity in the general population (Fooladi et al., 2020; Lippi et al., 2020; Yasui et al., 2020). Metal ions are crucial during viral infections, both for the survival of the virus and for the activation of the host immune system, which depends on the presence of micronutrients to maintain the integrity of its functions (Lukác and Massányi, 2007; Chasapis, 2018; Dharmalingam et al., 2021; Wessels et al., 2021). Additionally, it is well recognized that both metal deficiency and overload lead to abnormal cellular function or damage (Becker and Skaar, 2014). From a clinical perspective, alterations in the concentration of some metals may result in increased or decreased susceptibility to infection (Weiss and Carver, 2018).

During the SARS-CoV-2 pandemic, special attention has been given to the role of metal ions in the host response to infection and their interaction with the immune system (De Jesus and De Araújo Andrade, 2020; Dharmalingam et al., 2021). For example, serum Fe^3+^ concentration is inversely correlated with COVID-19 severity both before and after treatment, predicts the progression from mild and moderate to severe and critical illness, and is associated with the elevation of the inflammatory cytokine IL-6 (Hippchen et al., 2020; Sonnweber et al., 2020). Severe COVID-19 infection has also been associated with systemic Zn^2+^ depletion in the general population (Gonçalves et al., 2021; Skalny et al., 2021; Yasui et al., 2020, Vogel-González et al., 2021) and in pregnant women (Anuk et al., 2021), and this element is required to activate immune cells that regulate cell-mediated immunity (Hirano et al., 2008; Wessels et al., 2021). High serum Cu^2+^ concentration is also related to survival in COVID-19 (Hackler et al., 2021) being associated with low levels of Zn^2+^ in infected patients (Skalny et al., 2021), including pregnant women (Anuk et al., 2021). Indeed, it has been suggested that an increased serum Cu^2+^/Zn^2+^ ratio could be a valuable severity marker in COVID-19 patients (Anuk et al., 2021), as has been previously proposed for case-control studies with bacterial, viral, and parasitic infections (Van Weyenbergh et al., 2004; Kassu et al., 2006). Unlike the consistent findings for metals such as Fe^3+^, Zn^2+^, and Cu^2+^, serum levels of Na^+^, K^+^, Ca^2+^, and Mg^2+^ varied less consistently in COVID-19 patients (Frontera et al., 2020; Anuk et al., 2021; Skalny et al., 2021; Taheri et al., 2021; Van Kempen and Deixler, 2021; Yang et al., 2021). Although inflammatory responses and changes in serum metal concentration have been reported in COVID-19, few associations between metal ions and cytokines have been described in the general population or pregnant women. This study aimed to evaluate correlations between inflammatory markers and serum metal ions in third-trimester pregnant women with COVID-19 and their association with disease severity.

## Material and Methods

### Study Design

The present prospective study included third-trimester pregnant women who delivered at the National Institute of Perinatology and Hospital General de México Dr. Eduardo Liceaga in Mexico City, Mexico, from July 2020 to March 2021. The study population included SARS-CoV-2 negative women as a control group (*n* = 34) and SARS-CoV-2 positive women divided into asymptomatic (*n* = 76), mild (*n* = 64), and severe cases (*n* = 23). Because some of the markers measured in this study were outside the detection range of some test or the sample was insufficient, the number (n) of patients tested for each marker is indicated in the figure legends.

The asymptomatic group and healthy controls were attended at the National Institute of Perinatology, whereas all symptomatic patients were from the General Hospital of Mexico Dr. Eduardo Liceaga. Diagnosis of SARS-CoV-2 infection was based on a positive RT-qPCR to detect viral genome from nasopharyngeal swabs at least 24–48 h before delivery. Demographic data, comorbidities, laboratory results, symptoms, hospitalization status, and other clinical information were obtained from the medical record (Supplementary Table S1).

### Metal Ion Analysis

Blood samples were collected at delivery using BD Vacutainer^®^ Trace Element Serum Tubes by the medical staff. Blood was centrifuged at 3,500 rpm for 5 min and serum was then aliquoted and stored at –80°C until testing. Three different volumes (10, 20, and 30 µL) of serum were transferred to Eppendorf plastic microtubes containing 200 µL of concentrated Ultrapure nitric acid (Merck). The tubes were sealed and incubated at 60°C for 24 h to digest the material, and 1 ml of miliQ water was added to dilute the samples. Elemental analysis (Zn^2+^, Cu^2+^, Na^+^, K^+^, Fe^3+^, Ca^2+^, Mg^2+^, and P) was performed using a PerkinElmer Optima 8300 ICP-OES instrument (Shelton, CT, United States) with the appropriate calibration curves and a digestion blank containing Ultrapure nitric acid alone. Elemental analysis by ICP-OES does not distinguish oxidation states in the case of transition metal ions, but blood serum is an oxidizing environment where Cu^+^ and Fe^2+^ ions are not favored. Furthermore, the method does not distinguish between labile or tightly protein-bound ions. The values for phosphorus (P) represent an indirect measure of phosphates, including also, in this case, both the free ions and the attached phosphate moieties to other biomolecules.

Ionized Ca^2+^ and Mg^2+^ were analyzed using an ion-selective electrode (ISE) containing a neutral carrier-based membrane (Resnick et al., 1993).

Fingernail samples were collected using stainless steel nail clippers and were stored separately in labeled polyethylene bags. Fingernails were soaked and stirred in distilled water for 1 h, then dried in an oven at 60°C for 45 min. Twenty mg of sample was digested in 1 ml Ultrapure nitric acid at 200°C for 15 min in closed vessels of the MARS6 microwave digestion system (CEM Corporation, Matthews, NC, United States), as we previously described (Vásquez-Procopio et al., 2020). Samples were diluted with water to 5 ml and metal concentrations were measured as described above.

Placental tissue and umbilical cord samples were washed in distilled water, placed in 15 ml tubes, and kept at -80°C overnight; the samples were freeze-dried (48 h for placenta and 72 h for umbilical cord). From this point, the digestion and metal measurements followed the same procedure as with the fingernails detailed above.

### Serum Iron Markers

Iron biomarkers were quantified in blood serum using commercially available kits, according to the manufacturer’s instructions, at the Nutrition Laboratory of the Instituto Nacional de Perinatología. Ferritin was measured using enzyme-linked immunosorbent assay (ELISA) revealed by chemiluminescence (Immulite1000, Siemens, NY, United States). Soluble transferrin receptor (sTfR) (R&D Systems, Minneapolis, MN, United States), and bioactive hepcidin-25 (DRG-Diagnostics kit, Marburg, Germany) were quantified with colorimetric ELISAs (Flores-Quijano et al., 2019).

### Cytokine Quantification

The concentration of cytokines IL-1α, IL-1β, IL-1ra, IL-2, IL-4, IL-6, IL-8 IL-10, IL-12 (p40), IL-12 (p70), IL-17A, IP-10, CCL3, CCL4, TNF-α, eotaxin, and VEGF was measured in serum using the MILLIPLEX^®^ MAP Kit Human Cytokine/Chemokine Magnetic Bead Panel (96 well) (Millipore Corporation), according to the protocol provided by the manufacturer. A standard curve was used, and the data were acquired and analyzed on a Luminex platform using the Bio-Plex System and the Bio-Plex Manager software (Bio-Rad Laboratories, Hercules, CA) (Castro-Leyva et al., 2012). All cytokines were expressed as pg/ml.

### Statistics

Statistical analyses were performed using GraphPad Prism 9.1.2, Stata Statistical Software Release 17; Stata Corp LLC and R version 4.1.1. Continuous variables were described as mean ± standard deviation, while categorical variables were presented as percentage of prevalence (Table 1). The Shapiro test was applied to evaluate normality. For normally distributed data, the one-way ANOVA test was used to compare between groups, and Pearson’s correlation test was used for correlation analysis. For non-normal distributions, the Kruskal-Wallis test was used for comparisons between groups and Spearman’s correlation for correlation analysis. The Chi-squared test was used to compare the categorical variables. Cytokine and metal ion data were used to predict logistic regression models to estimate the probability of being severe COVID-19 during pregnancy. After logistic regression, the adjusted model’s performance was evaluated using a receiver operating characteristic curve (ROC) analysis, estimating the area under the curve and determining the best cut-off value of biochemical markers for the predictions of severe COVID-19 in pregnant women.

**TABLE 1:** Demographic, laboratory, and clinical variables of SARS-CoV-2 positive pregnant women and healthy controls.

Characteristic or Clinical Condition	Control *n* = 34	Asymptomatic *n* = 76	Mild *n* = 64	Severe *n* = 23	*p* value
Maternal age, mean ± SD	29.0 ± 5.7	30.2 ± 6.8	30.7 ± 5.8	30.5 ± 5.4	0.655
Gestational age, mean ± SD	38.1 ± 0.9	38.2 ± 1.6	37.0 ± 3.1	33.2 ± 4.2	<0.0001
Body mass index, mean ± SD	27.3 ± 5.0	27.3 ± 6.6	28.4 ± 5.2	26.3 ± 5.5	0.097
Hypertension, n (%)	3 (9%)	4 (5%)	5 (9%)	1 (4%)	0.852
Preeclampsia, n (%)	2 (6%)	2 (3%)	4 (6%)	3 (15%)	0.200
Diabetes, n (%)	1 (3%)	6 (8%)	0 (0%)	0 (0%)	0.325
Gestational diabetes, n (%)	4 (12%)	4 (5%)	7 (11%)	3 (13%)	0.507
Hypothyroidism, n (%)	1 (3%)	12 (16)	7 (11%)	1 (4%)	0.179
Hemoglobin (g/dl), mean ± SD	12.0 ± 1.6	12.5 ± 1.7	12.4 ± 1.7	12.0 ± 1.7	0.575
Hematocrit %, mean ± SD	36.4 ± 4.7	38.0 ± 4.7	37.2 ± 5.3	37.3 ± 5.2	0.380
Erythrocyte (mill/mm3), mean ± SD	4.1 ± 0.6	4.2 ± 0.5	4.2 ± 0.5	4.2 ± 0.6	0.880
Leukocytes (mill/mm3), mean ± SD	9.1 ± 2.1	8.7 ± 2.3	8.9 ± 2.9	10.0 ± 4.0	0.284
Platelets (mill/mm3), mean ± SD	236 ± 55	250 ± 78	227 ± 68	207 ± 63	0.045
Neutrophil %, mean ± SD	70.7 ± 8.9	68.7 ± 8.5	76.0 ± 10.8	83.4 ± 9.5	<0.0001
Lymphocyte %, mean ± SD	21.2 ± 8.0	22.6 ± 7.8	16.2 ± 7.7	10.7 ± 6.6	<0.0001
Triglycerides (mg/dl), mean ± SD	—	—	296 ± 110	275 ± 100	0.576
Cholesterol (mg/dl), mean ± SD	—	—	212 ± 57	152 ± 43	<0.0001
Myoglobin (ng/ml), mean ± SD	—	—	26.1 ± 17.5	44.7 ± 34.7	0.030
D Dimer (ng/ml), mean ± SD	—	—	2,929 ± 1710	2,436 ± 2,741	0.027
C reactive protein (mg/L), mean ± SD	—	—	47 ± 67	112 ± 90	<0.0001
Procalcitonin (ng/ml), mean ± SD	—	—	0.24 ± 0.52	0.46 ± 0.43	0.0001
SatO2%, mean ± SD	—	—	94.1 ± 6.5	88.6 ± 8.3	<0.0001
SpO2%, mean ± SD	—	—	90.9 ± 13.1	87.9 ± 8.1	0.021
IUC admission, n (%)	—	—	1 (2%)	11 (48%)	<0.0001
Fever, ≥ 37.3, n (%)	—	—	19 (30%)	17 (74%)	<0.0001
Fatigue, n (%)	—	—	24 (38%)	17 (74%)	<0.0001
Cough, n (%)	—	—	28 (44%)	21 (91%)	<0.0001
Dyspnea, n (%)	—	—	10 (16%)	16 (70%)	<0.0001
Myalgia, n (%)	—	—	18 (28%)	21 (91%)	<0.0001
Chest paint, n (%)	—	—	5 (8%)	13 (57%)	<0.0001
Pneumonia, n (%)	—	—	7 (1%)	19 (83%)	<0.0001
Maternal death, n (%)	—	—	0 (0%)	3 (15%)	0.003

Markers were described as numbers and proportions in contingency tables. For the linear discriminant analysis, 30 patients of the control group, 53 asymptomatic, 37 mild, and 16 severe patients with all cytokine and metal ion data available, were analyzed with the MASS package. 3D graphics were visualized utilizing the Scatterplot3d package with R.

## Results

### Demographic and Clinical Characteristics of Third-Trimester Pregnant Women With COVID-19

We studied 197 pregnant women who delivered in two third-level reference hospitals in Mexico City from July 2020 through March 2021. All subjects were tested for SARS-CoV-2 infection by RT-qPCR in nasopharyngeal swabs 24–48 h before delivery. Women with a positive test were classified into asymptomatic (*n* = 76) and symptomatic (*n* = 87) groups based on the absence of symptoms or the presence of at least one of the symptoms consistent with COVID-19 (Chen N. et al., 2020; Garg et al., 2020; Hu et al., 2021). For symptomatic patients, the severity of COVID-19 was assessed according to oxygen saturation level (SatO2), the need for an extended period of hospitalization (unrelated to the pregnancy), intensive care unit admission, mechanical ventilation, or maternal death (Gandhi et al., 2020; Kucirka et al., 2020). Thus, 23 pregnant women were considered in the severe group, while the remaining 64 were included in the mild group characterized by minor symptoms. A healthy control group of women with a negative test was also evaluated (*n* = 34).

The demographic and clinical data of the studied population are summarized in Table 1 and fully available in.(Supplementary Table S1) No differences were found in maternal age, body mass index, and preexisting medical conditions such as hypertension or diabetes, gestational diabetes, preeclampsia, or hypothyroidism between the groups. Pregnant women in the severe COVID-19 group were characterized by neutrophilia (*p* < 0.0001), lymphopenia (*p* < 0.0001), and thrombocytopenia (*p* = 0.045) when compared to the other three groups, accompanied by increased concentrations of acute phase reactants such as CRP (*p* < 0.0001), D dimer (*p* = 0.027), and procalcitonin (*p* = 0.0001) compared to the mild group. Additionally, myoglobin was elevated in women included in the severe group (*p* = 0.030), while cholesterol levels were lower (*p* < 0.0001) than those with the mild form of COVID-19.

Women with severe COVID-19 had a higher prevalence of preterm delivery (78.3%), twice as much as women in the mild group (34.8%) (*p* = 0.038). Preterm birth was due to severe deterioration in maternal health because of the infection. There were no stillbirths among women with SARS-CoV-2 during pregnancy, and 3 women in the severe group died.

### Serum Cytokine Profile in Third-Trimester Pregnant Women With COVID-19

**FIGURE 1:**
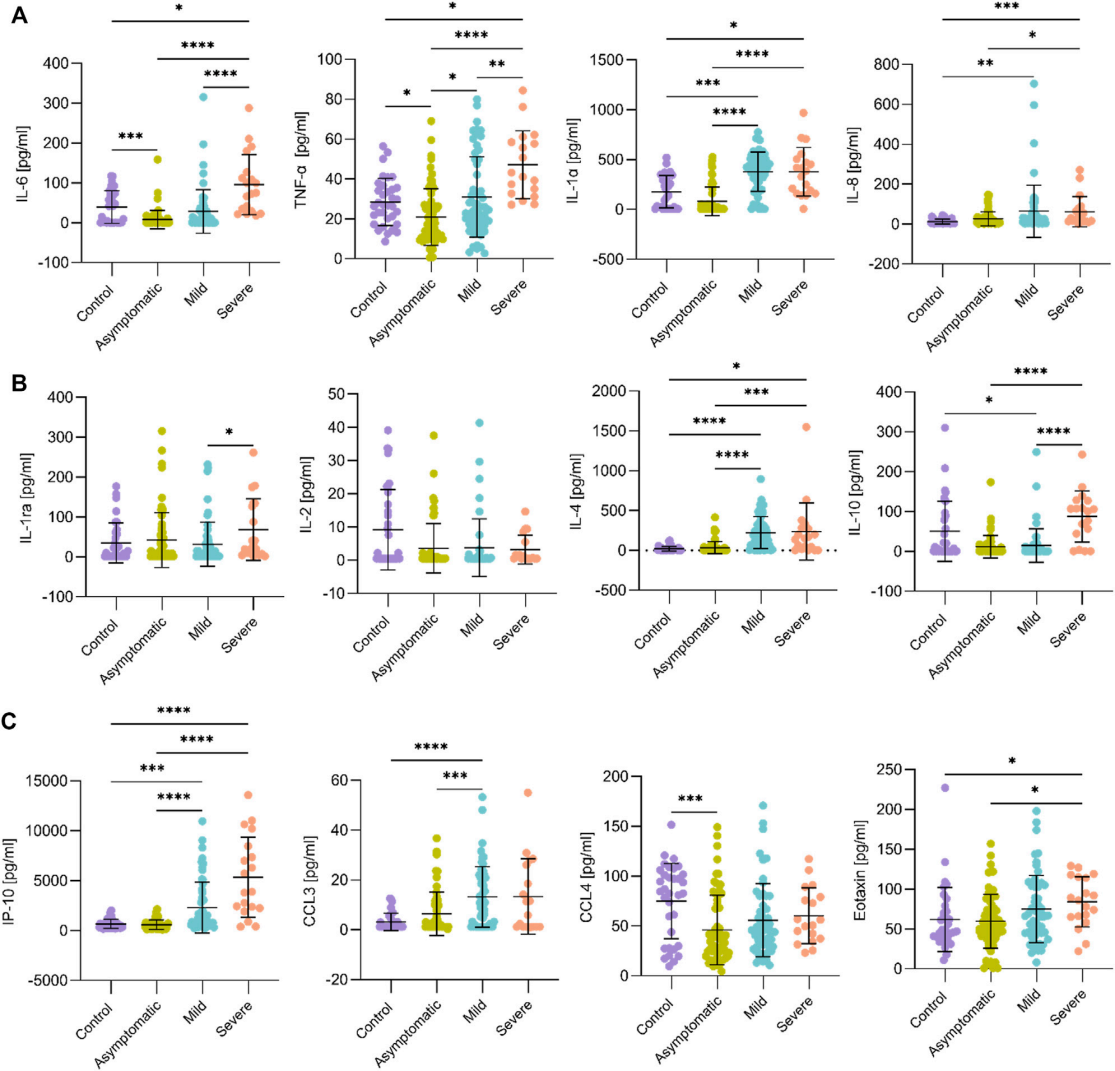
Comparison of serum cytokine and chemokine levels in third trimester pregnancy and in relation to SARS-CoV-2 infection and the severity of associated symptoms. **(A,B)**. Data from the indicated cytokines from control (*n* = 34; purple), asymptomatic (*n* = 67; green), mild (*n* = 58; blue), and severe (*n* = 19; orange) patients were plotted. Pro-inflammatory cytokines IL-6, TNF-, IL-1α and IL-8 increased in symptomatic women with COVID-19 versus the control group, while IL-2 and IL-1ra did not change. Anti-inflammatory cytokine IL-4 was also increased in symptomatic women with COVID-19 versus the control group. Note that IL-6, TNF-, and IL-1α decreased in the asymptomatic group. **(C)**. Similar plots for selected chemokines. IP-10 was markedly higher in women with mild and severe symptoms compared to uninfected patients (control group), whereas eotaxin was increased only in the latter (severe group). CCL4 was lower in the asymptomatic group. Standard deviations of the mean are shown in all panels. Comparisons between groups were performed using the Kruskal-Wallis test. Asterisks denote, respectively, *p < 0.05, **p < 0.01, ***p < 0.001, ****p < 0.0001.

Maternal blood samples were collected at delivery to determine 17 serum cytokines using the Pro Human Cytokine Milliplex Panel (Castro-Leyva et al., 2012). IL-6 and TNF-α were significantly increased in the severe group compared to the other groups (*p* < 0.05) (Figure 1A). Other pro-inflammatory cytokines that were induced in the mild and severe groups compared to healthy controls were IL-8 (*p* < 0.001) and IL-1α (*p* < 0.05) (Figure 1A). Cytokines such as IL-2, IL-1β, IL-17, IL-12p70, IL-12p40, and VEGF were unaffected during SARS-CoV-2 infection in pregnant women (Figure 1B, Supplementary Figure S1).

Anti-inflammatory cytokines IL-4 and IL-10 were also studied (Figure 1B). IL-10 concentration was increased (*p* < 0.001) in the severe group compared to mild and asymptomatic groups, similar to IL-6 and TNF-α. Furthermore, IL-4 was tenfold higher in women with mild and severe symptoms compared to the control and asymptomatic groups (*p* < 0.05). Regarding chemokines, only IP-10 was increased two-fold in the mild group (*p* < 0.001) and seven-fold in the severe group (*p* < 0.0001) compared to controls (Figure 1C). The rest of the cytokines (CCL3, CCL4, and eotaxin) showed non-significant changes between the groups (Figure 1C).

### Metal Ion Dysregulation in Third-Trimester Pregnant Women With COVID-19

**FIGURE 2:**
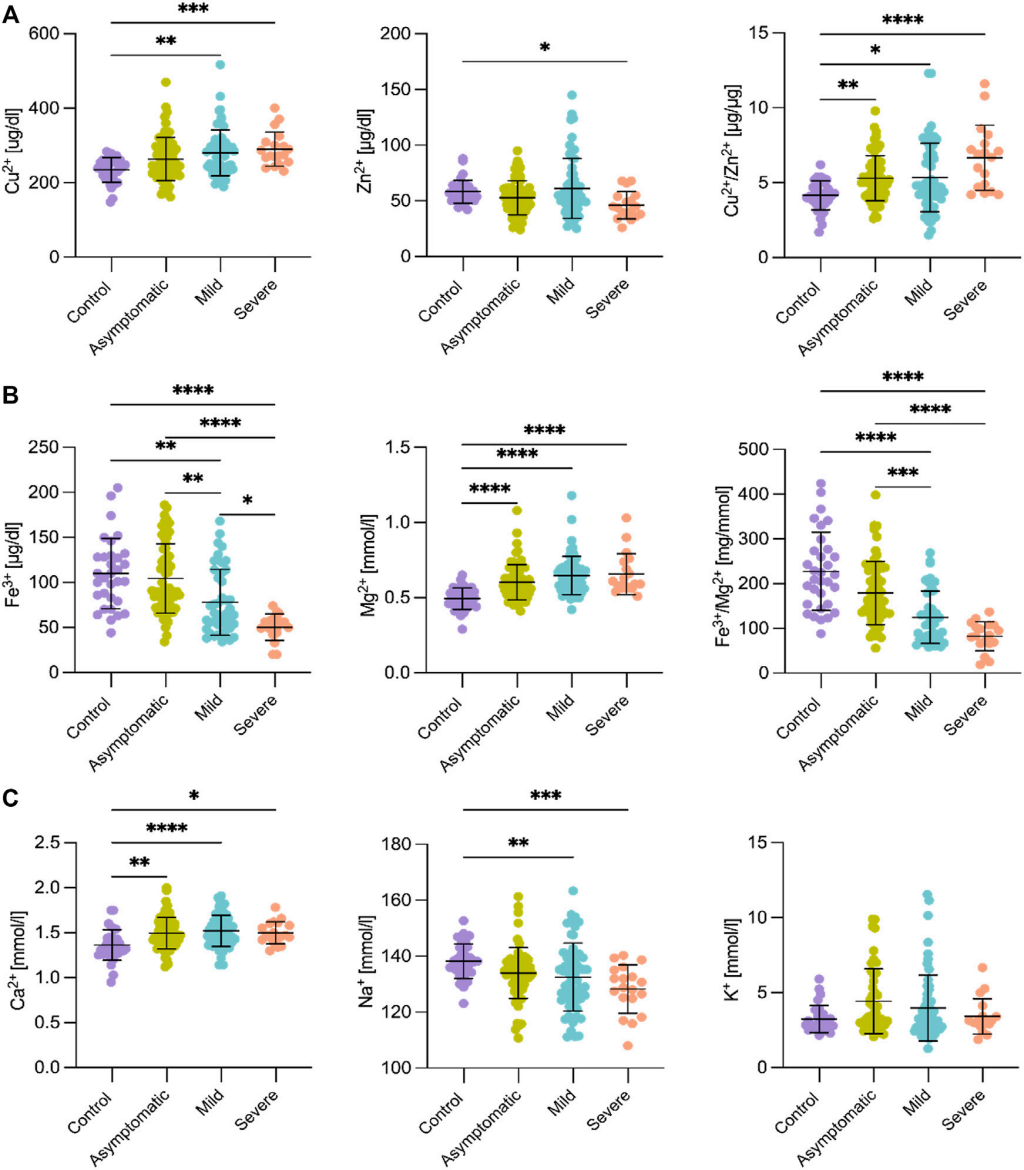
Serum metal ion concentrations in third trimester pregnant women infected with SARS-CoV-2. **(A)** Cu^2+^ increased in symptomatic (mild and severe) COVID-19 women, while Zn^2+^ decreased in the severe group by comparing with healthy pregnant patients. The Cu^2+^/Zn^2+^ ratio separated all COVID-19 patients from the control group. **(B)** Fe^3+^ decreased, while Mg^2+^ increased according to COVID-19 illness compared to control. The Fe^3+^/Mg^2+^ ratio discriminated between all different groups. **(C)** Ca^2+^ increased with SARS-CoV-2 infection, Na^+^ decreased in women with mild and severe symptoms and K^+^ was unaffected. Control (*n* = 34), asymptomatic (*n* = 69), mild (*n* = 61) and severe cases (*n* = 19). Standard deviations of the mean are shown in all panels. Comparisons between groups were performed using Kruskal-Wallis test. Asterisks denote, respectively, *p < 0.05, **p < 0.01, ***p < 0.001, ****p < 0.0001.

Blood samples collected at delivery were also analyzed using inductively coupled plasma optical emission spectroscopy (ICP-OES) to determine serum concentrations of Cu^2+^, Zn^2+^, Fe^3+^, Mg^2+^, Na^+^, K^+^, Ca^2+^, and P (Figure 2, Supplementary Figure S2, and Supplementary Table S1). The concentration of Cu^2+^ was significantly higher in women with mild and severe symptoms (*p* < 0.001) compared to healthy controls (Figure 2A), while Zn^2+^ concentration was lower in the severe group (*p* < 0.05). The Cu^2+^/Zn^2+^ ratio discriminated infected patients from the control group (Figure 2A).

**FIGURE 3:**
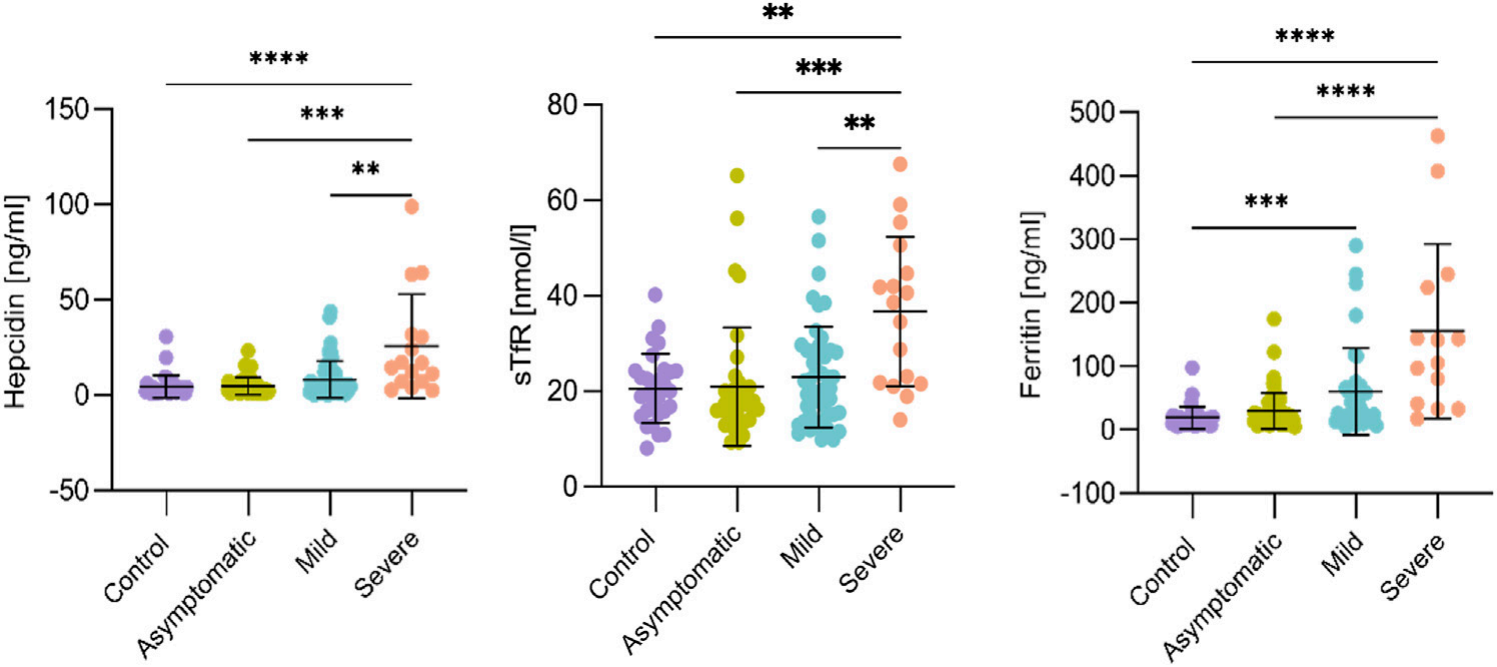
Serum iron markers in third trimester pregnant patients with COVID-19. Serum iron parameters are excellent markers of the severe form of COVID-19 symptomatology. Standard deviations of the mean are shown in all panels. Hepcidin and soluble transferrin receptor (sTfR) increased solely in the group of women with severe symptoms compared to all other groups, while serum ferritin was higher in both the mild and severe groups compared to the control group. For control, *n* = 34; Asymptomatic, *n* = 64; Mild, *n* = 57; Severe, *n* = 16. Comparisons between groups were performed using the Kruskal-Wallis test. Asterisks denote, respectively, *p < 0.05, **p < 0.01, ***p < 0.001, ****p < 0.0001.

Iron status was also altered in pregnant women infected with SARS-CoV-2. The serum concentration of Fe^3+^ was lower in the mild (*p* < 0.01) and the severe (*p* < 0.001) forms of the disease compared to the control group (Figure 2B). Moreover, Fe^3+^ showed statistically significant differences (*p* < 0.05) between the severe group and all other three groups, but also against the mild and asymptomatic or control groups. In pregnant women with COVID-19, a significantly higher concentration of hepcidin (*p* < 0.01) was found in women in the severe group (Figure 3), with the highest concentration in one of three women who died (Supplementary Table S1). sTfR levels were significantly higher in the severe COVID-19 women compared to the other groups (*p* < 0.01), while serum ferritin concentration was elevated in both the mild (*p* < 0.001) and severe groups (*p* < 0.0001) compared to the control group (Figure 3).

In contrast to the low Fe^3+^ level, women with SARS-CoV-2 infection showed significantly higher serum Mg^2+^ concentration compared to uninfected women, as well as to the mild and asymptomatic forms of the disease (*p* < 0.0001 for all comparisons) (Figure 2B). In addition, we measured serum ionized Mg^2+^ using an ion-selective electrode, verifying the increased presence of Mg^2+^ ions in pregnant women with COVID-19 by an independent method (Supplementary Figure S2). We also calculated the Fe^3+^/Mg^2+^ ratio, which discriminated very robustly between symptomatic and asymptomatic pregnant women (Figure 2B; right panel).

About the rest of the elements, serum Ca^2+^ concentration was significantly higher (*p* < 0.05) in COVID-19 pregnant women regardless of the severity of the disease. Conversely, Na^+^ concentration was lower (*p* < 0.01) in symptomatic patients versus the control group (Figure 2C). Two elements, K^+^ (Figure 2C) and P (Supplementary Figure S2), showed no differences between pregnant women infected with SARS-CoV-2 and healthy controls, which differs from the general population.

### Metal Homeostasis in Fingernails, Placenta, and Umbilical Cord

**FIGURE 4:**
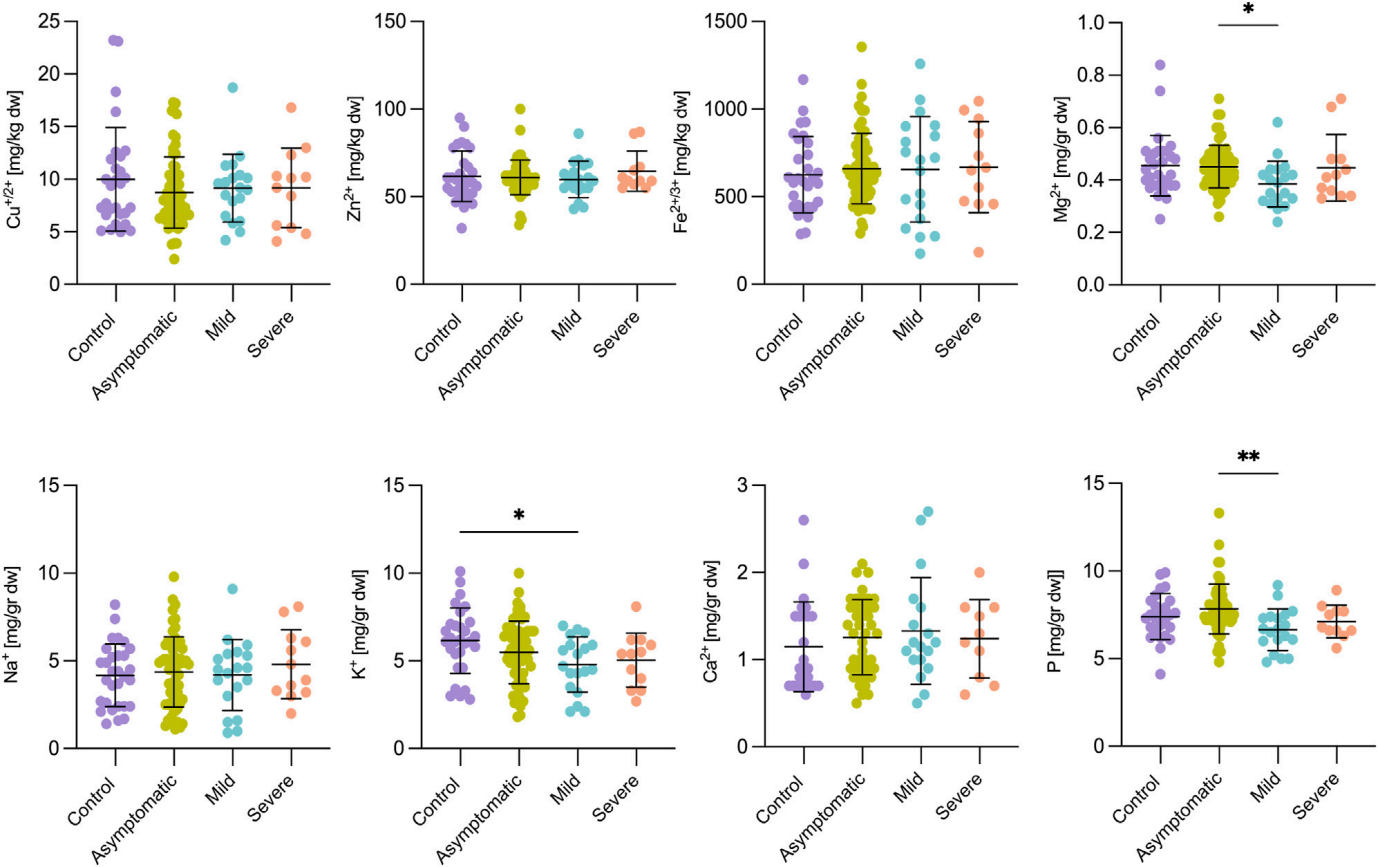
Metal ion concentrations were largely unaffected in the placenta of women infected with SARS-CoV-2. Standard deviations of the mean are shown in all panels. For the control group, *n* = 30; Asymptomatic, *n* = 65; Mild, *n* = 21; Severe, *n* = 10. One-way ANOVA was run for normally distributed data (K^+^ and Na^+^) and the Kruskal-Wallis test otherwise (Zn^2+^, Cu^+^/^2+^, Fe^2+^/^3+^, Mg^2+^, P and Ca^2+^). Asterisks denote, respectively, *p < 0.05, **p < 0.01.

Fingernail Zn^2+^, Cu^2+^, Mg^2+^, Na^+^, and Fe^3+^ ions were not significantly different between the four studied groups (Supplementary Figure S3), and symptomatic patients (mild *p* < 0.5; severe *p* < 0.001) presented a significantly higher level of fingernail Ca^2+^ compared to the control group. The level of P in fingernails was higher in the severe patients compared to the control group (*p* < 0.05), although serum levels of P were unchanged between these groups. K^+^ ions showed a more dynamic pattern of change in fingernails, with a noticeable drop in the asymptomatic infected group compared to the control (*p* < 0.05) and severe (*p* < 0.001) groups (Supplementary Figure S3). When comparing the placenta (Figure 4) or umbilical cord (Supplementary Figure S4), no change was observed in any of the metals in the control and severe groups.

### Inflammatory Response and Serum Metallome Correlation

**FIGURE 5:**
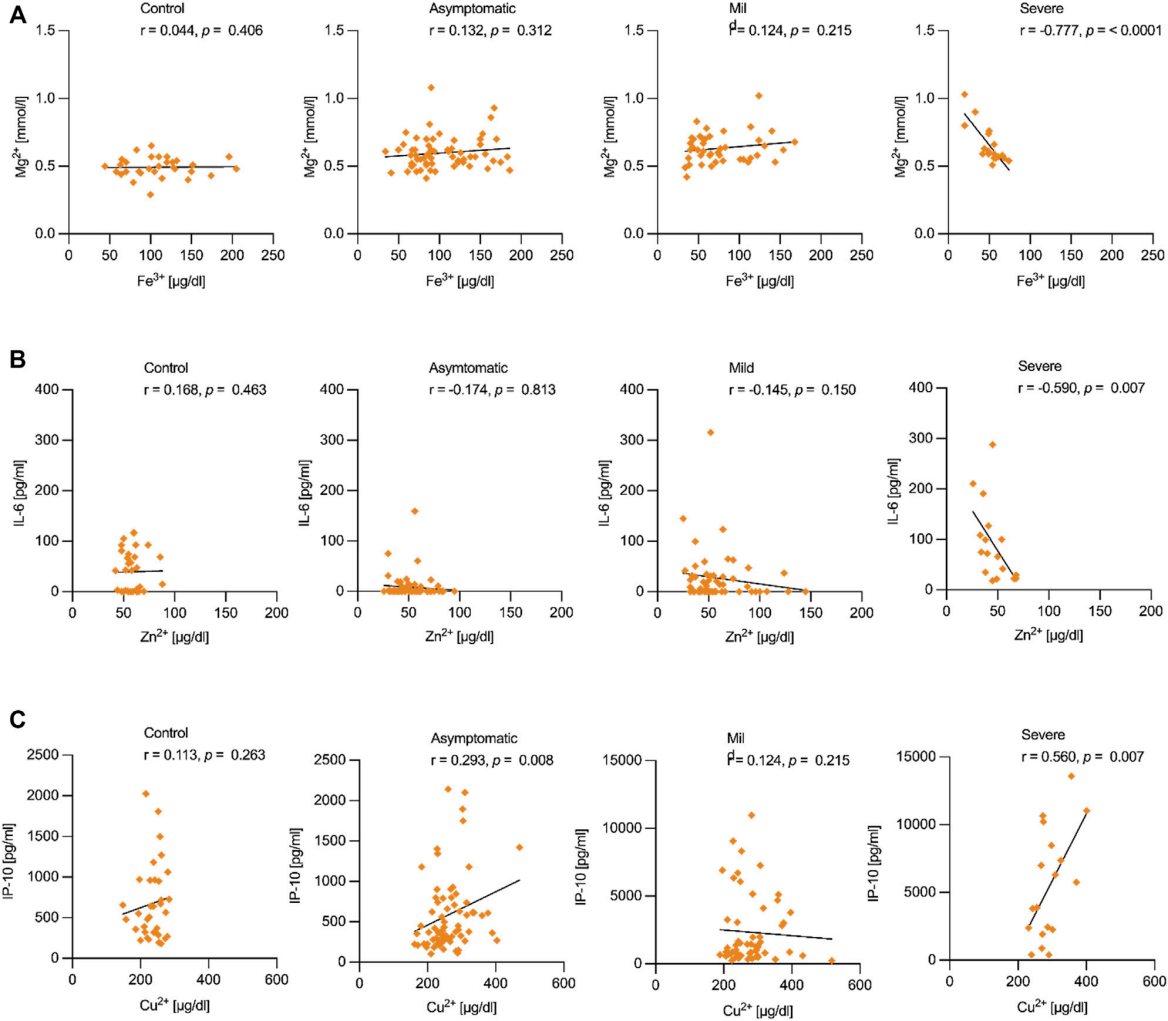
Correlations between metal ions and cytokines in pregnant women with severe COVID-19. **(A)**. The values for serum Fe^3+^ and Mg^2+^ ions were plotted for each patient within the four groups of patients included in the present study. A negative correlation (r = -0.777, *p* < 0.001) was present only in the severe COVID-19 group. **(B)**. As above, the values for Zn^2+^ and IL-6 were plotted and a negative correlation (r = -0.590, *p* = 0.007) was determined in pregnant women with severe symptoms. **(C)**. A positive correlation (r = 0.560, *p* = 0.007) between serum Cu^2+^ ions and the IP-10 chemokine IP-10 was also observed in severe COVID-19. The type of correlation analysis considered whether the data were distributed normally; Pearson’s coefficient was run for normally distributed data (Cu^2+^ and IP-10) and Spearman’s coefficient otherwise (Fe^3+^ and Mg^2+^, and Zn^2+^ and IL-6). Control (*n* = 34), asymptomatic (*n* = 67), mild (*n* = 58) and severe cases (*n* = 19).

After investigating how cytokines and metal ions were affected in pregnant women infected with SARS-CoV-2, we performed a correlation analysis to evaluate associations. We analyzed within each group of patients, revealing three sets of robust correlations (Figure 5). Fe^3+^ and Mg^2+^ ions were negatively correlated (r = -0.777, *p* < 0.001), specifically in the group of women with severe symptoms (Figure 5A). Indeed, we found that the Fe^3+^/Mg^2+^ ratio was positively related to SatO2 (Supplementary Figure S5A), implicating the two metals in the severe form of COVID-19 illness. SatO2 was inversely correlated with IL-6 (Supplementary Figure S5B), a major inflammatory cytokine that also correlated negatively with serum Zn^2+^ ions (r = -0.590, *p* = 0.007) in the severe group of patients (Figure 5B). In other words, the subset of patients that responded to the infection by secreting higher levels of IL-6 tended to show lower levels of serum Zn^2+^ ions. Again, this negative correlation was only observed in the severe group (Figure 5B). The third correlation (r = 0.560, *p* = 0.007) was a positive association between Cu^2+^ ions and IP-10 identified in women with severe COVID-19 (Figure 5C). CRP also correlated positively with Cu^2+^ (r = 0.691, *p* = 0.001) in the severe, but not in the mild group (Supplementary Figure S5C).

Cu^2+^ ions did not correlate with Zn^2+^ in the population studied here; however, the Cu^2+^/Zn^2+^ ratio was positively associated with neutrophils (r = 0.578 *p* = 0.006) and leukocytes (r = 0.595, *p* = 0.004), and negatively with lymphocytes (r = -0.597, *p* = 0.004) in pregnant women with severe COVID-19 (Supplementary Figure S5C). suggesting that Cu^2+^/Zn^2+^ ratio is a more useful measure than the serum concentration of Cu^2+^ or Zn^2+^ ions alone.

### Biochemical Markers and Disease Severity

**FIGURE 6:**
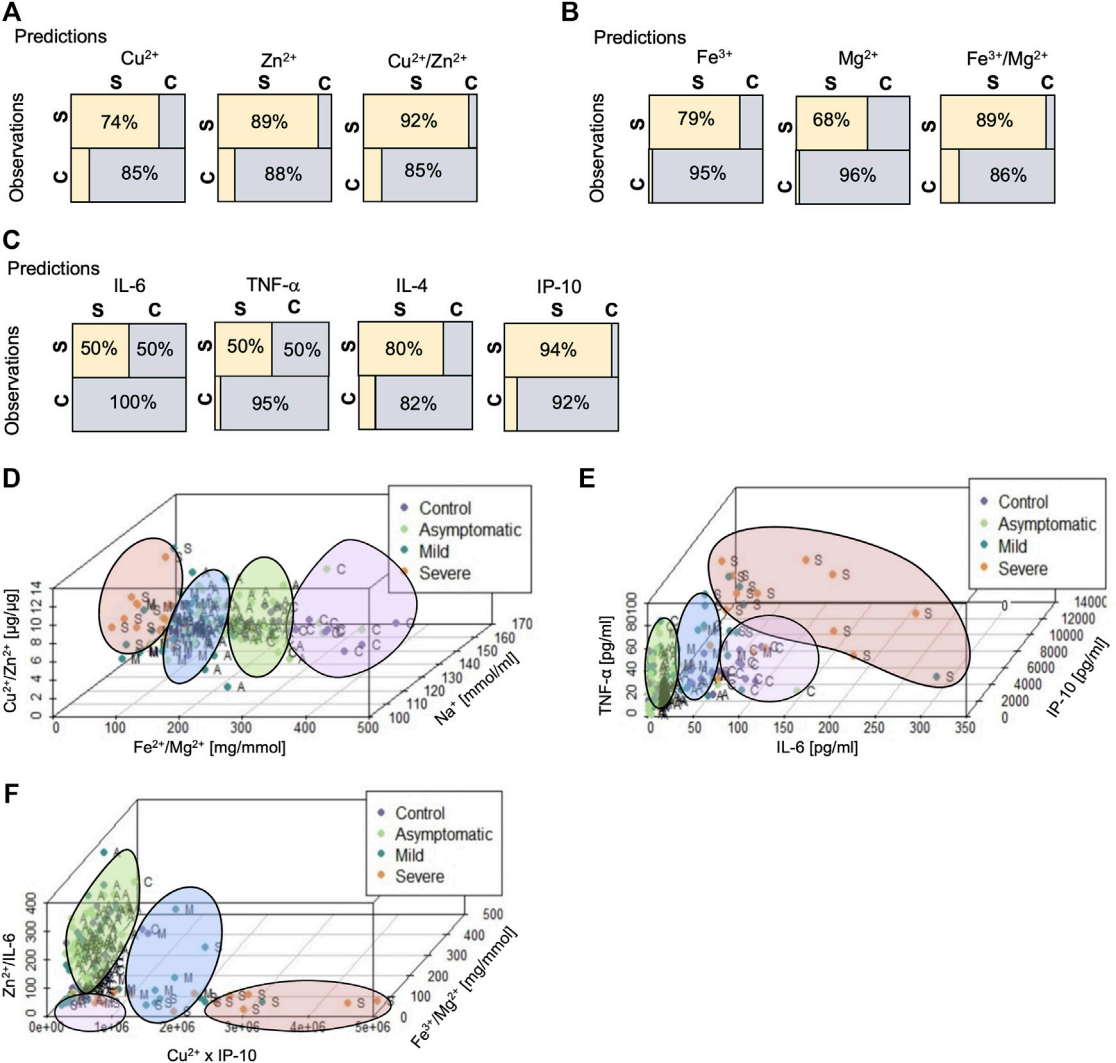
Application of metal ion and cytokine biomarkers to evaluate the presence and severity of SARS-CoV-2 infection in pregnancy. **(A–C)**. Visual representation of the confusion matrix derived from predicted and observed values calculated from a ROC analysis of the indicated biochemical markers in patients belonging to the control (C) or severe (S) groups where the rows represent predicted categories, and the columns represent actual categories. Note the added value of using the Cu^2+^/Zn^2+^ and Fe^3+^/Mg^2+^ ratios as correlates of severe COVID-19. **(D–F)**. 3-dimensional (3D) plot for visual representation of the different variables of all study groups. Individuals from the control group (*n* = 30) are shown in purple, asymptomatic patients (*n* = 53) are shown in green, patients with mild symptoms (*n* = 37) in blue, and patients with severe COVID-19 (*n* = 16) in orange. Capitalized initial letters for each group on the side of each data point correspond to the prediction offered by the linear discriminant analysis model. The four groups of pregnant women are broadly concentrated on different spaces indicated by the colored illustrations. See Supplementary Table S3 for quantification and comparison of the effectiveness of the linear discriminant models.

To investigate whether specific metal ions or cytokines whose concentrations differed between healthy and pregnant women with severe COVID-19 could be used to segregate efficiently between the two groups, we performed a ROC analysis and applied the best cut-off value to predict severe COVID-19 in pregnant women. Details of these calculations are presented in the supplementary information (Supplementary Table S2). Using the values for serum Cu^2+^, 14 of 19 (74%) patients in the severe group were allocated correctly (Figure 6A). In the case of serum Zn^2+^ ions, the categorization was 16 of 18 (89%) pregnant women. However, when the Cu^2+^/Zn^2+^ ratio was applied, 12 of 13 (92%) severe patients were correctly predicted (Figure 6A). Our study revealed that Fe^3+^ serum concentration also correlated well with disease progression. Used as a predictor, serum Fe^3+^ correctly identified 15 out of 19 patients (79%) in the severe group (Figure 6B), whereas serum Mg^2+^ detected 13 out of 19 patients (68%). The ratio Fe^3+^/Mg^2+^ correctly recognized 17 out of 19 patients (89%) with the severe form of the disease (Figure 6B).

To set the above findings in context, we performed a similar analysis with the inflammatory cytokines IL-6 and TNF-α, the anti-inflammatory cytokine IL-4, and the chemokine IP-10. Use of IL-6 or TNF-α correctly identified 8 out of 16 patients (50%) of the severe group, IP-10 identified 16 out of 17 patients (94%), whereas IL-4 only misplaced three infected women (80%) (Figure 6C).

The Cu^2+^/Zn^2+^ and Fe^3+^/Mg^2+^ ratios properly discriminated between the groups when plotted against the Na^+^ values (Figure 2). In the resulting 3-dimensional (3D) plot (Figure 6D), two aspects are evident: 1) the model generates four groups of patients, and 2) there is a continuum of values with some mismatching of individuals. These mismatches mainly were observed between adjacent groups (i.e., control versus asymptomatic) but lowered the overall predictive capacity of the linear discrimination models (Supplementary Table S3). When this type of analysis was performed using IP-10, IL-6, and TNF-α (Figure 6E) as well as the Fe^3+^/Mg^2+^ and Zn^2+^/IL-6 ratios, and the product Cu^2+^ x IP-10 (Figure 6F), we found that metal ions alone were 50% successful in correctly assigning patients to the respective groups, cytokines were 54%, and the combined model was 52% successful (Supplementary Table S3).

### Personalized Observations of Cytokine/Metal Concentrations in Maternal Deaths

We analyzed separately the cytokine and metal parameters of the 3 cases that resulted in maternal death comparing the mean values calculated for all patients in the severe group. Interestingly, all three patients showed concentrations above 385 pg/ml for IL-1α and above 5,484 pg/ml for IP-10, with values below 50 μg/dl for serum Fe^3+^ and below 46 μg/dl for serum Zn^2+^ ions, which was not observed in women with severe disease who survived, regardless of whether they presented pneumonia or were admitted to the IUC (Supplementary Table S4).

## Discussion

In this work, we provide evidence that immune and serum metal ion responses in third-trimester pregnant women infected with SARS-CoV-2 vary according to the severity of the disease. Clinical manifestations in pregnant women with COVID-19 range from asymptomatic to severe (Sevilla-Montoya et al., 2021); furthermore, some studies have described that the infection triggers a peripheral maternal inflammatory response (Zhang et al., 2017; Cornish et al., 2020; Goldstein et al., 2020), and changes in metal ion concentrations (Kilbride et al., 1999; Sangaré et al., 2014). Here, we have bridged these observations by studying the systemic cytokine and metal ion profile in asymptomatic and symptomatic women, and including a more significant number of severe cases than in previous reports.

Since the onset of the COVID-19 pandemic, it was established that patients who progressed to more severe forms of the disease shared some clinical and laboratory features (Zinellu et al., 2021); therefore, the detailed description of the women included in our study was fundamental, dividing the symptomatology into moderate and severe to describe more accurately the changes in cytokines and metal ions and associate them with the severity of the infection. In addition to the triad neutrophilia-lymphopenia-thrombocytopenia and increased concentrations of CRP, D dimer, and procalcitonin, myoglobin was found to be elevated in patients with severe COVID-19, which correlates with a hypoxemic state (Ma et al., 2021). Consistent with what has been described by other authors (Hu et al., 2020; Wei et al., 2020; Zinellu et al., 2021), pregnant women in the severe group had lower cholesterol levels (*p* < 0.0001) than those in the mild group; although this finding has no pathophysiologic explanation so far, it has been proposed that cholesterol concentrations might be helpful, in combination with other clinical and demographic variables, for risk stratification and monitoring in patients with the severe forms of the disease (Zinellu et al., 2021). Remarkably, pregnant women with risk factors such as obesity, diabetes, and hypertension were not especially affected by the severe form of COVID-19, in contrast to what is known for the general population (Garg et al., 2020; Guan et al., 2020; Killerby et al., 2020; Sanyaolu et al., 2020).

COVID-19 is currently considered an inflammatory disease, and it is well known that the severe forms present an exacerbated inflammatory response, the cytokine storm, which plays a major role in the clinical evolution of the patients and frequently results in multisystemic end-organ damage (Chen G. et al., 2020; Tung-Chen et al., 2021). Accordingly, in our work, we observed that key inflammatory markers (IL-6, TNF-α, IL-8, IL-1α, and IP-10) were elevated in serum from hospitalized women due to severe features, such as pneumonia. Moreover, IL-1α seems to have a pivotal role in the induction of cytokine storm due to uncontrolled immune responses in COVID-19 infection (Mardi et al., 2021). IP-10, which has been proposed as an early identifier of lung disease in COVID-19 patients (Howe et al., 2021), was increased two-fold in the mild group and seven-fold in the severe group compared to controls. Interestingly, only TNF-α increased gradually as disease severity increased, as reported before (Ben Moftah and Eswayah, 2022). On the other hand, lower circulating levels of IL-6, TNF-α, IL-α, and CCL4 were observed in the asymptomatic group of pregnant women with COVID-19 than healthy pregnant women. In the general population, asymptomatic SARS-CoV-2 infection did not affect these cytokines (Long et al., 2020), but pregnancy is known to affect cytokines independently of COVID-19 (Marzi et al., 1996). The reasons for cytokine suppression in asymptomatic pregnant women infected by the virus remain unclear. Our results differ from a recent study in which the determination of cytokines in maternal plasma was also performed and found no significant differences between the control group with infection and women with COVID-19 confirmed by RT-PCR positive for SARS-CoV-2, except for IL-15 (Garcia-Flores et al., 2022). This discrepancy could be explained by the smaller number of patients that were included (n = 7) and the fact that asymptomatic patients (n = 5) and patients with moderate (n = 1) and severe (n = 1) symptoms were considered within the same group, also mentioning that they found no differences in the clinical characteristics between them.

We found that serum metal ions were significantly affected in pregnant women with COVID-19: Zn^2+^, Fe^3+^, and Na^+^ decreased, whereas Ca^2+^, Mg^2+^, and Cu^2+^ increased according to disease severity. Serum Cu^2+^ ion raises during pregnancy, independently of infection, and is doubled at full term (Vukelić et al., 2012). High Cu^2+^ ion was recently reported in another study of pregnant women with SARS-CoV-2 (Anuk et al., 2021). In the general population, individuals who survived SARS-CoV-2 infection also increased the major serum Cu^2+^ binding protein ceruloplasmin (Hackler et al., 2021). Ceruloplasmin is presumably secreted from the liver into the bloodstream to maintain iron homeostasis (Roeser et al., 1970; Vasilyev, 2010). During pregnancy, physiologic iron demands increase considerably to support fetoplacental development and maternal adaptation to pregnancy, which may result in iron deficiency (Fisher and Nemeth, 2017). Serum Fe^3+^ ions concentration decreased in SARS-CoV-2 infected patients and was directly related to the severity of COVID-19, in concordance to what is known for the general population (Sonnweber et al., 2020; Zhao et al., 2020; Girelli et al., 2021). However, this reduction in iron levels is not exclusive to SARS-CoV-2 infection, as it is also observed in ICU patients, as wells as in patients with sepsis, chronic heart failure, chronic kidney disease, and inflammatory bowel disease, among others (Patteril et al., 2001; Cappellini et al., 2017).

Besides low serum Fe^2+^ ions, the severe group of patients showed high serum ferritin, hepcidin, and sTfR concentrations, consistent with the characteristic pattern of anemia associated with inflammation (Ueda and Takasawa, 2018; Ratnaningsih et al., 2020). It is well established that inflammation affects systemic iron homeostasis and its master regulator, hepcidin (Nemeth et al., 2003; Ueda and Takasawa, 2018); consistently, hepcidin is also released during inflammation arising from the SARS-CoV-2 infection (Zhou et al., 2020; Nai et al., 2021). High serum ferritin is frequently observed in hyperinflammation, with a worse prognosis and higher mortality in hospitalized COVID-19 patients (Hippchen et al., 2020; Girelli et al., 2021).

How metals are regulated at the systemic level is not well understood, except for iron regulation (Anderson and Shah, 2013; Tejeda-Guzmán et al., 2018; Chen J. et al., 2020; Garay et al., 2022). When analyzing the data for associations between the different biochemical parameters, we unexpectedly identified a strong negative correlation between Fe^3+^ and Mg^2+^, where serum Fe^3+^ ions deficiency correlated with increased serum Mg^2+^, but only in patients presenting a severe reaction to COVID-19. Notably, others have also reported an increase in Mg^2+^ concentration in pregnant women with COVID-19 (Anuk et al., 2021) and high levels of serum Mg^2+^ ions were observed in kidney transplant patients positive to *Helicobacter pylori* (Hafizi et al., 2014) and with visceral leishmaniasis infectious disease (Lal et al., 2013) indicating a possible role of Mg^2+^ in infectious illness. Increased serum Mg^2+^ ions could either be a cause of renal failure (Azem et al., 2020) or a physiological attempt to induce the adaptive immune response through the metal’s binding on the co-stimulatory cell-surface molecule LFA-1, which requires Mg^2+^ to adopt its active conformation on CD8^+^ T cells (Lötscher et al., 2022).

We also discovered that the increase of Cu^2+^ levels correlated positively with IP-10, a chemokine that exerts potent biological functions (Liu et al., 2011) and is highly associated with disease severity and progression in COVID-19 (Yang et al., 2020). A positive correlation between Cu^2+^ and IP-10 has not been previously reported, possibly because it appeared exclusively in patients with severe symptomatology, as seen with the negative correlation between Fe^3+^ and Mg^2+^. In this study, the severe form of the disease during pregnancy was associated with a third negative correlation, as women with high levels of IL-6 tended to be Zn^2+^ deficient. In other words, the subset of patients that responded to the infection by secreting more elevated levels of IL-6 tended to show lower levels of serum Zn^2+^ ions. Again, this negative correlation was only observed in the severe group and the result corroborates a previous finding in COVID-19 pregnant women (first trimester) that associated high IL-6 with low levels of serum Zn^2+^ (Anuk et al., 2021). Earlier studies have documented the association between serum Zn^2+^ ions deficiency and COVID-19 (Gonçalves et al., 2021; Skalny et al., 2021; Vogel-González et al., 2021) (Yasui et al., 2020), including studies in pregnant women (Anuk et al., 2021). These findings are consistent with the known Zn^2+^ requirement in maintening of immune functions (Read et al., 2019; Wessels et al., 2021). The present study adds two points of crosstalk between cytokines/chemokines and serum metal ions in the subset of pregnant women who were severely affected by the SARS-CoV-2 infection: IL-6 with Zn^2+^ (Foster and Samman, 2012; Jung et al., 2015; Wong et al., 2021), and IP-10 with Cu^2+^.

Before this work, the Cu^2+^/Zn^2+^ ratio had been proposed as an indicator of SARS-CoV-2 infection (Anuk et al., 2021; Skalny et al., 2021). The Cu^2+^/Zn^2+^ ratio has been suggested as a potential biomarker of inflammation and mortality in an elderly population and has been associated with a higher risk of infectious diseases leading to hospitalization (Malavolta et al., 2010; Laine et al., 2020). Interestingly, our work complements and corroborates previous studies, showing that the Cu^2+^/Zn^2+^ ratio was higher in pregnant women infected with SARS-CoV-2 and may be able to predict 92% of the individuals with severe COVID-19 as a binary marker. Furthermore, the ratio Fe^3+^/Mg^2+^ introduced here showed 89% successful prediction of severe COVID-19. More studies should be conducted to determine whether it can serve as a useful clinical marker. Notably, three biochemical parameters, the ratio Zn^2+^/IL-6, the product Cu^2+^ x IP-10, and the Fe^3+^/Mg^2+^ ratio, could be used to segregate pregnant women into the control, asymptomatic, mild, and severe groups.

Although we did not find a characteristic profile of cytokines, metals or the combination of both in maternal death, severe pneumonia or ICU admission, it is important to highlight the fact that all patients who did not survive presented a cytokine storm characterized by IL-1a and IP-10 levels above the severe group mean and at the same time, Zn^2+^ and Fe^3+^ levels below the severe group mean. This affected the Cu^2+^/Zn^2+^ and Fe^3+^/Mg^2+^ ratios and also the ratio Zn^2+^/IL-6 and the product Cu^2+^ x IP-10, which may indicate that there are some cytokine-metal connections involved in the clinical evolution that triggered death that should be studied further. The fact that we did not find obvious patterns with the variables we measured in the study could be due to the small number of maternal deaths included or that the cause of death is multifactorial, which would make difficult the prognostic characterization of COVID-19 in pregnancy.

In addition to maternal serum, the concentration of metal ions in the fingernails was also determined, as previous reports have proposed this measure as a potential bioindicator for longer-term storage for some elements (Mehra and Juneja, 2005), including heavy metals (Goullé et al., 2009) (Hussein Were et al., 2008). Fingernail Zn^2+^, Cu^2+^, and Fe^3+^ ions were not significantly different between the four studied groups, suggesting that the significant serum alterations in the transition metal ions were most likely the result of the patients’ acute immunological response to the SARS-CoV-2 infection. Likewise, serum changes in Mg^2+^ and Na^+^ were probably also SARS-CoV-2 infection outcomes, because fingernail Mg^2+^ and Na^+^ remained unchanged between the groups. In contrast, symptomatic patients presented a significantly higher level of fingernail Ca^2+^ compared to the control group, which is consistent with the increase in Ca^2+^ concentration observed in serum. These results suggest a dynamic deposition pattern for the latter three elements, in contrast to transition metal ions.

Essential elements play critical roles in fetal growth and development, and imbalances in metal ions are associated with a higher risk of fetal malformation and preterm birth (Gambling and McArdle, 2004; Terrin et al., 2015; Grzeszczak et al., 2020). We, therefore, isolated placental and proximal umbilical cord tissues to measure ions at the interface between mother and fetus. Interestingly, no change was observed in any metals when comparing the placenta or umbilical cord in the control and severe groups. Thus, although COVID-19 significantly altered serum metal ions, placental and umbilical cord metal homeostasis remained functional.

Overall, the main aim of this study was to determine which cytokines and metal ions were associated with COVID-19 severity during the third trimester of pregnancy, exploring correlations between them. Therefore, the most substantial finding in this work is that coordinated alterations occur between serum metal ions and cytokines in pregnant women with COVID-19, suggesting that underlying pathophysiological interactions between them could be involved in disease severity. The metal dysregulation observed in pregnant women with COVID-19 could alter many immunological processes where metal ions are required. For example, zinc deficiency has been shown to increase neutrophils and decrease lymphocytes (Wessels et al., 2022), whereas low blood iron also disrupts the progression of the adaptive immune response (Frost et al., 2021), consistent with our findings. Changes in metal ion concentrations during SARS-CoV-2 infections are therefore not random; instead, our study complements the work of others (De Jesus and De Araújo Andrade, 2020; Anuk et al., 2021; Skalny et al., 2021) to show the biological significance of metal ions in the mounting of a successful immune response against viral infections. We conclude that alterations in metal ion and cytokine concentrations are evidence of immune failure and severity in pregnant women with COVID-19.

## Data Availability

The original contributions presented in the study are included in the article/Supplementary Material, further inquiries can be directed to the corresponding authors.
