# Supplementary material for: Inflammatory-Metal Profile as a Hallmark for COVID-19 Severity During Pregnancy
Source: Front Cell Dev Biol. 2022 Aug 9;10:935363. doi: 10.3389/fcell.2022.935363 (PMC9395991; doi:10.3389/fcell.2022.935363)
Supplement: Supplementary file 1 [file Table2.docx]

**Supplementary Table 2. Receiver operating characteristic (ROC) calculations of the indicated biochemical markers in patients belonging to the Control (C) or Severe (S) groups.**

| **Biochemical markers** | **Cutpoint** | **AUC** | **Estimate** | **Specificity** | **95% IC** |
| --- | --- | --- | --- | --- | --- |
| Copper_serum | 268 µg/dL | 0.79 | 73.7% | 58.2% | 0.676-0.913 |
| Zinc_serum | 68 µg/dL | 0.50 | 11.1% | 88.2% | 0.415-0.596 |
| Cu/Zn ratio | 5.6 µg/ µg | 0.81 | 66.7% | 97.9% | 0.702-0.934 |
| Fe_serum | 70 µg/dL | 0.89 | 94.4% | 84.4% | 0.810-0.978 |
| Mg_serum | 0.54 mmol/L | 0.84 | 94.7% | 73.5% | 0.750-0.932 |
| Fe/Mg | 95 mg/mmol | 0.76 | 55.6% | 96.9% | 0.640-0.884 |
| IL-6 | 17.89 pg/ml | 0.74 | 100% | 48.5% | 0.655-0.829 |
| TNF-a | 27.29 pg/ml | 0.73 | 94.7% | 52.9% | 0.638-0.837 |
| IL-4 | 62.66 pg/ml | 0.77 | 63.2% | 91.1% | 0.650-0.893 |
| IP-10 | 1908.37 pg/ml | 0.90 | 84.2% | 97.0% | 0.817-0.995 |
