# Supplementary material for: Inflammatory-Metal Profile as a Hallmark for COVID-19 Severity During Pregnancy
Source: Front Cell Dev Biol. 2022 Aug 9;10:935363. doi: 10.3389/fcell.2022.935363 (PMC9395991; doi:10.3389/fcell.2022.935363)
Supplement: Supplementary file 3 [file Table3.docx]

**Supplementary Table 3. Errors in classification of patients to their corresponding group according to the Linear Discriminant Analysis models presented in the main text.**

| **METALLOME BASED PREDICTION** |  |  |  |  |
| --- | --- | --- | --- | --- |
| **Overall error: 50%** | **Predicted Control** | **Predicted Asymptomatic** | **Predicted Mild** | **Predicted Severe** |
| Real Control | 10 (33%) | 18 | 2 | 0 |
| Real Asymptomatic | 8 | 38 (68%) | 9 | 1 |
| Real Mild | 0 | 17 | 15 (42%) | 4 |
| Real Severe | 0 | 1 | 9 | 6 (37%) |
| **CYTOKINE BASED PREDICTION** |  |  |  |  |
| **Overall error: 46%** | **Predicted Control** | **Predicted Asymptomatic** | **Predicted Mild** | **Predicted Severe** |
| Real Control | 9 (30%) | 19 | 2 | 0 |
| Real Asymptomatic | 3 | 52 (93%) | 1 | 0 |
| Real Mild | 3 | 21 | 4 (11%) | 8 |
| Real Severe | 2 | 0 | 4 | 10 (63%) |
| **COMBINED INDICATOR BASED PREDICTION** |  |  |  |  |
| **Overall error: 48%** | **Predicted Control** | **Predicted Asymptomatic** | **Predicted Mild** | **Predicted Severe** |
| Real Control | 11 (37%) | 19 | 0 | 0 |
| Real Asymptomatic | 10 | 40 (71%) | 6 | 0 |
| Real Mild | 0 | 16 | 13 (36%) | 7 |
| Real Severe | 0 | 2 | 6 | 8 (50%) |
