# Supplementary material for: Inflammatory-Metal Profile as a Hallmark for COVID-19 Severity During Pregnancy
Source: Front Cell Dev Biol. 2022 Aug 9;10:935363. doi: 10.3389/fcell.2022.935363 (PMC9395991; doi:10.3389/fcell.2022.935363)
Supplement: Supplementary file 5 [file Image1.pdf]

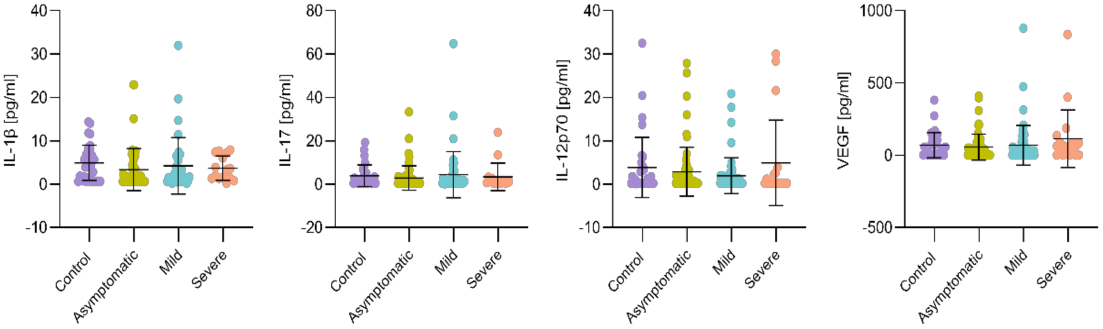

Supplementary Figure 1. Cytokine levels in third trimester pregnant women infected with SARS-CoV-2. IL-1β, IL-17, IL-12p70 and VEGF-A were unaffected by the infection. Standard deviations of the mean are shown in all panels. n = 34 in the Control group, n = 67 for Asymptomatic, n = 58 for Mild, and n = 19 for the Severe group. Comparisons between groups were performed using the Kruskal-Wallis test.

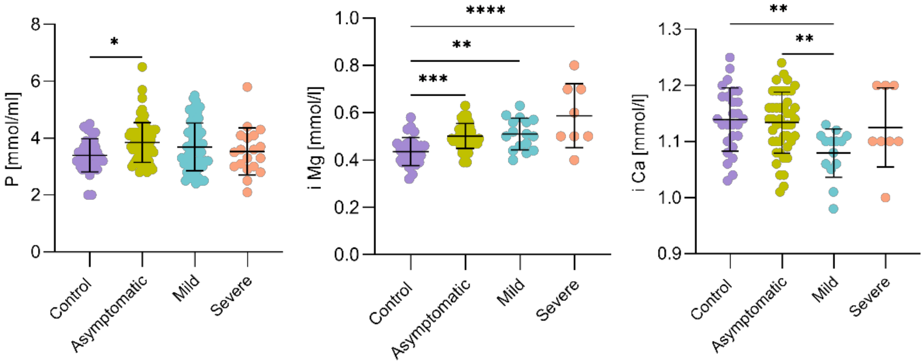

Supplementary Figure 2. Serum phosphorus (P), ionized magnesium (iMg), and ionized calcium (iCa) concentrations in sera of third trimester pregnant women grouped according to COVID-19 disease severity. Standard deviations of the mean are shown in all panels. A slight increase in P was observed in the Asymptomatic group when compared to the Control group of patients. Kruskal-Wallis test was run for these data with n = 34 for the control group, n = 69 for asymptomatic, n = 60 for mild, and n = 19 for severe. iMg was higher in all groups of patients infected with SARS-CoV-2. This finding correlates with the observed increase total serum magnesium (Figure 2B). In contrast, iCa decreased in the group of women with mild symptoms, compared to healthy or asymptomatic patients, a result which is different to the measurements of total serum Ca^{2+} (Figure 2C), suggesting the additional total Ca^{2+} observed in infected patients is protein-bound. For the latter assays the data were distributed normally and therefore one-way Anova was used. The control group had n = 29, asymptomatic had n = 51, mild had n = 15, and severe had n = 9. Asterisks denote, respectively, * $p < 0.05$, ** $p < 0.01$, *** $p < 0.001$, **** $p < 0.0001$.

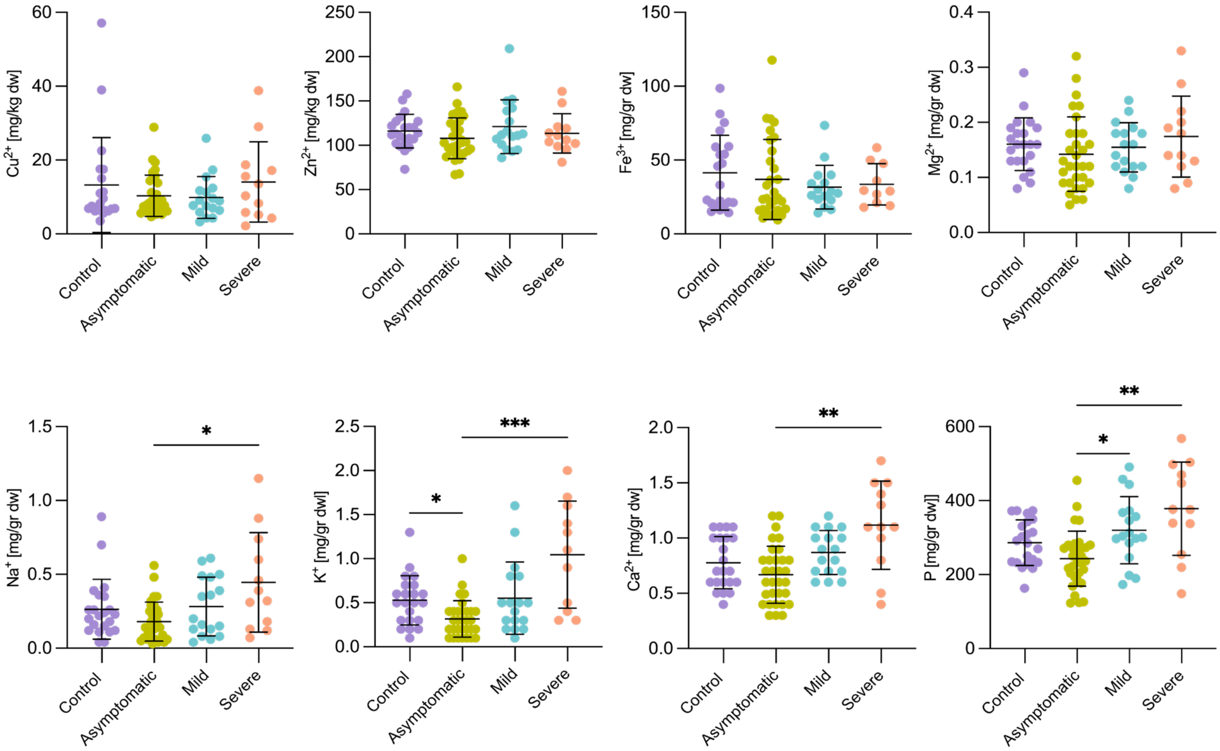

Supplementary Figure 3. Metal ion concentrations in fingernails from third trimester pregnant women with COVID-19. Standard deviations of the mean are shown in all panels. Cu^{2+}, Zn^{2+}, Fe^{3+}, Mg^{2+} and Na^{+} were unaffected by the infection. K^{+} levels were lower in asymptomatic infected women *versus* the control, but also *versus* the severe, groups. Ca^{2+} and P accumulated at higher levels in the severe group. Control, n = 22; asymptomatic, n = 31; mild, n = 18; severe, n = 11. One-way Anova was run for normally distributed data (P), while the Kruskal-Wallis test for the rest of the elements. Asterisks denote, respectively, * $p < 0.05$, ** $p < 0.01$, *** $p < 0.001$, **** $p < 0.0001$.

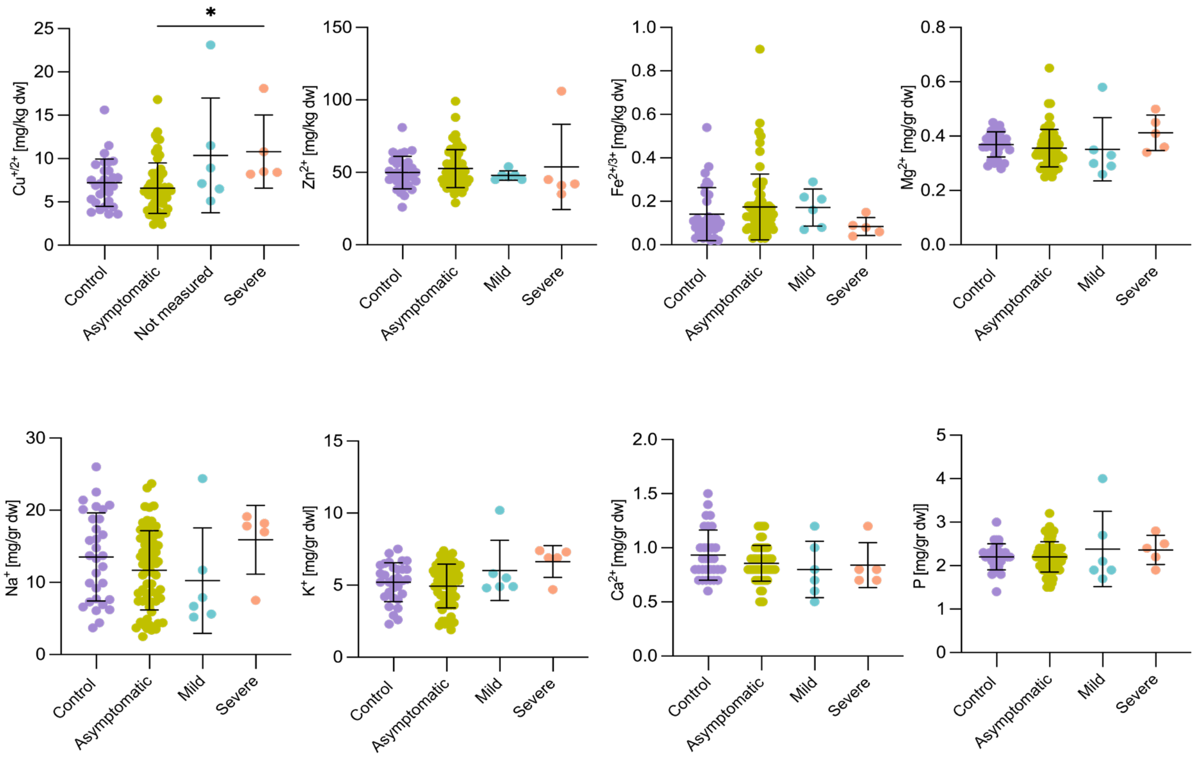

Supplementary Figure 4. Metal ion concentrations were unaffected in the umbilical cord of women infected with SARS-CoV-2. Standard deviations of the mean are shown in all panels. For the control group, n = 31; asymptomatic, n = 64; mild, n = 6; severe, n = 5. Comparisons between groups were performed using the Kruskal-Wallis test. * $p < 0.05$.

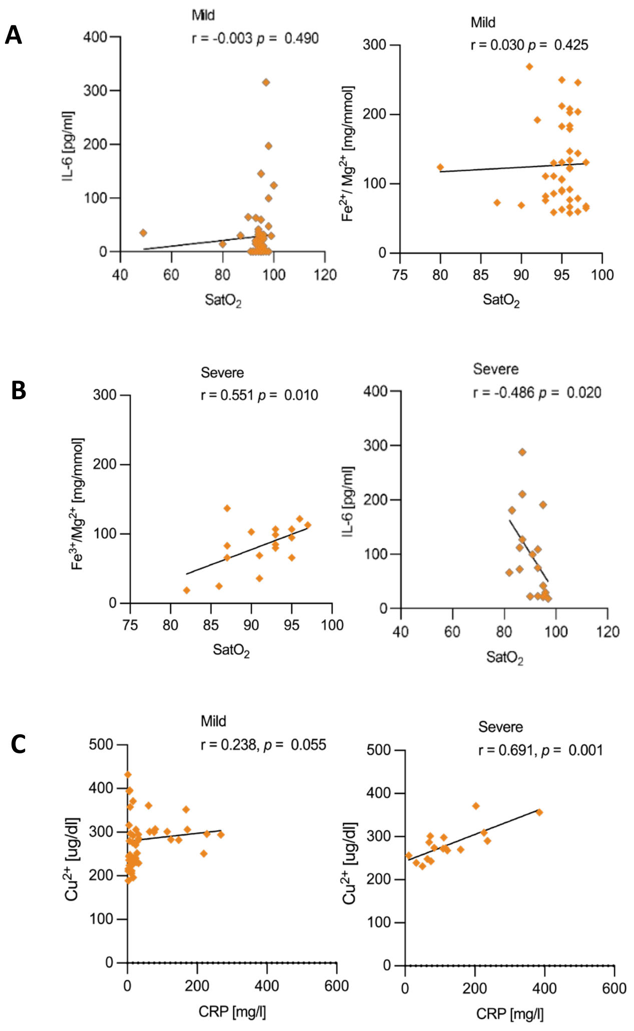

Supplementary Figure 5. Notable correlations between biochemical and clinical parameters in symptomatic patients with COVID-19. A. Oxygen saturation ($SatO_2$) was positively associated with the Fe^{3+}/Mg^{2+} ratio in the severe group of patients, suggesting that this biomarker correlates well with disease severity; Pearson's coefficient was used in this case, as data distributed normally. **B.** $SatO_2$ correlated negatively with IL-6 in women with severe symptoms also corroborating that higher IL-6 concentration is a marker of severe COVID-19. Spearman's coefficient is shown. **C.** C-reactive protein (CRP) correlated with copper (Cu^{2+}) in the severe group. Spearman's coefficient is shown. n = 58 for the mild group and n = 17 for the severe group.

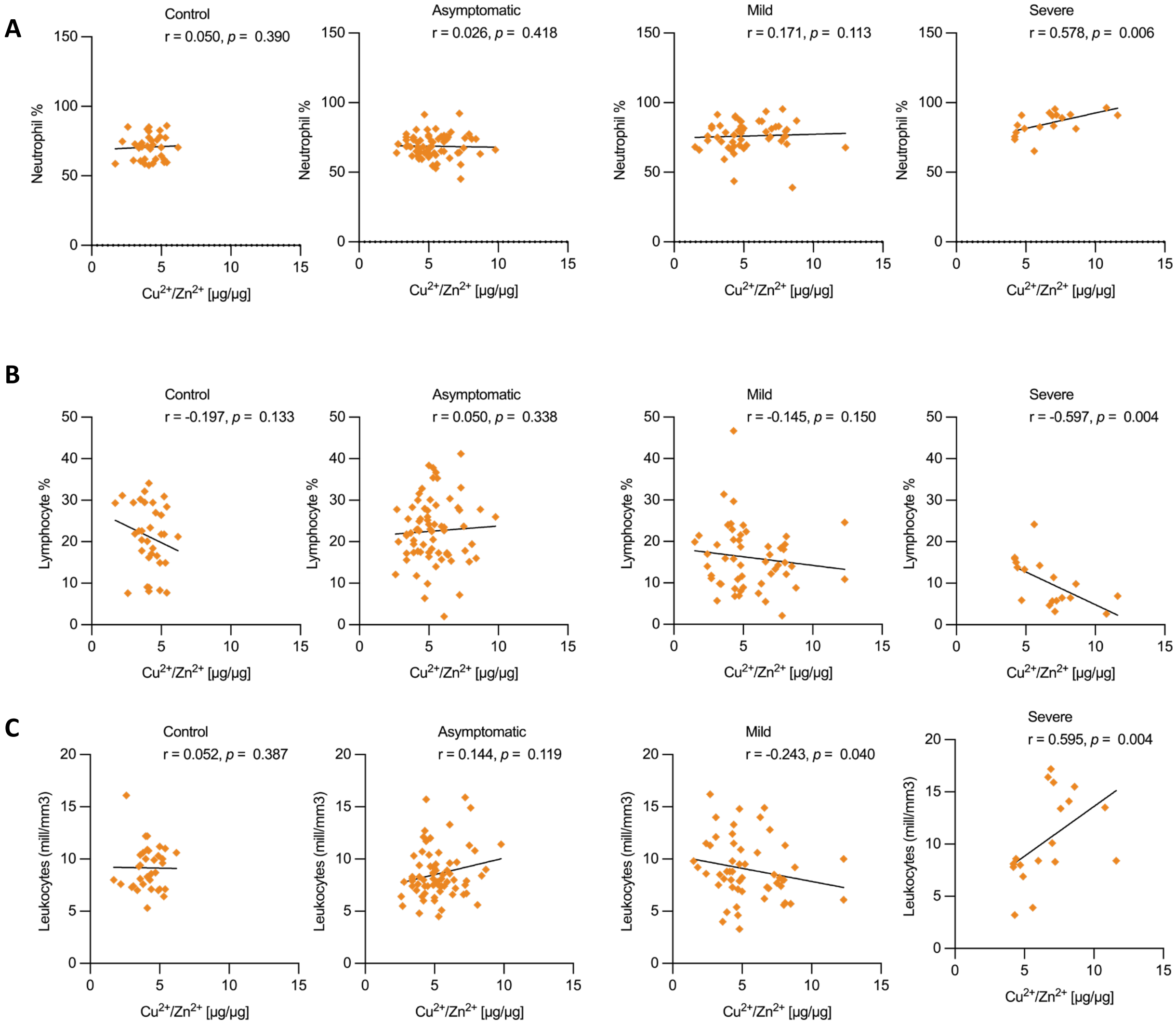

Supplementary figure 6. Correlations between the Cu^{2+}/Zn^{2+} ratio and immune cells. A. The Cu^{2+}/Zn^{2+} ratio associated positively with neutrophiles in the severe symptomatic group of pregnant women with COVID-19. **B.** In the same group of patients, the Cu^{2+}/Zn^{2+} ratio correlated negatively with lymphocytes. **C.** Consistently, a positive correlation between Cu^{2+}/Zn^{2+} ratio and leukocytes were observed in severe COVID-19. These results further corroborate the utility of the Cu^{2+}/Zn^{2+} ratio when monitoring the severity of COVID-19 during pregnancy. The type of correlation analysis considered whether the data were distributed normally; Pearson's coefficient was run for normally distributed data (Cu^{2+}/Zn^{2+} ratio and neutrophiles or lymphocytes) and Spearman's coefficient when the Cu^{2+}/Zn^{2+} ratio was related to leukocytes. n = 34 for Control, n = 67 for asymptomatic, n = 58 for mild, and n = 19 for severe cases.
